# Supplementary material for: Effectiveness of treat-to-target cholesterol-lowering interventions on cardiovascular disease and all-cause mortality risk in the community-dwelling population: a target trial emulation
Source: Nat Commun. 2024 Nov 15;15:9922. doi: 10.1038/s41467-024-54078-4 (PMC11568141; doi:10.1038/s41467-024-54078-4)

Supplementary information of

"Effectiveness of treat-to-target cholesterol-lowering interventions on cardiovascular disease and all-cause mortality risk in the community-dwelling population: a target trial emulation"

Zhao Yang, Qiujv Deng, Yongchen Hao, Na Yang, Lizhen Han, Pingping Jia, Pan Zhou, Yiming Hao, Ziyu Wang, Wenlang Zhao, Yue Qi, Jing Liu

Contents

Supplementary Tables..........2

Supplementary Table 1. Covariates used to model the risk of developing cardiovascular disease, all-cause mortality, and atherosclerotic cardiovascular disease..........2

Supplementary Table 2. Estimated parametric g-formula risk (%), risk difference (RD, %), and restricted mean event-free based number needed to treat (NNT) to prevent one cardiovascular disease, all-cause mortality, and atherosclerotic cardiovascular disease under natural course and treat-to-target interventions recommended by the 2020 Chinese Society of Cardiology (unless stated) after 29 years of follow-up from the Chinese Multi-provincial Cohort Study 1992-2020..........4

Supplementary Table 3. Estimated risk ratio (RR), restricted mean event-free time (RMET, years), and cumulative percentage of intervention under treat-to-target interventions recommended by the 2020 Chinese Society of Cardiovascular compared to the natural course of no interventions after 29-year follow-up from the Chinese Multi-provincial Cohort Study 1992-2020 *via* the parametric g-formula..........8

Supplementary Table 4. Estimated parametric g-formula risk (%), risk difference (RD, %), and restricted mean event-free based number needed to treat (NNT) to prevent one cardiovascular disease, all-cause mortality, and atherosclerotic cardiovascular disease under natural course and feasible interventions (unless stated) after 29 years of follow-up from the Chinese Multi-provincial Cohort Study 1992-2020..........12

Supplementary Table 5. Estimated risk ratio (RR), restricted mean event-free time (RMET, years), and cumulative percentage of intervention under feasible interventions compared to the natural course of no interventions after 29-year follow-up from the Chinese Multi-provincial Cohort Study 1992-2020 *via* the parametric g-formula..........15

Supplementary Figures..........18

Supplementary Figure 1. The observed (red dot) and predicted (blue line) levels of low-density lipoprotein cholesterol (LDL-C) using the generalized linear mixed model after accounting for age, sex, body mass index, hypertension, diabetes, and the use of cholesterol-lowering drugs for nine randomly selected participants (IDs 661, 1842, 1895, 3723, 4857, 4893, 15730, 35986, and 35993)..........18

Supplementary Tables

Supplementary Table 1. Covariates used to model the risk of developing cardiovascular disease, all-cause mortality, and atherosclerotic cardiovascular disease

Covariates	Years assessed	As outcomes	Functional form as covariates
**Time fixed covariates**			
Age at baseline	1992	Not predicted	Linear
Sex	1992	Not predicted	2 categories
Attained education	1992	Not predicted	2 categories
Occupation	1992	Not predicted	2 categories
**Time-varying covariates** [†]			
Smoking status	1992 to 2020 annually[†]	Logistic	2 categories
Body mass index	1992 to 2020 annually	Linear	Linear
Systolic blood pressure	1992 to 2020 annually	Linear	Linear
Total cholesterol	1992 to 2020 annually	Linear	Linear
Low-density lipoprotein cholesterol	1992 to 2020 annually	Linear	Linear
Triglyceridemic	1992 to 2020 annually	Linear, zero-inflated normal	Linear
Non-HDL-C	1992 to 2020 annually	Linear	Linear

Diabetes	1992 to 2020 annually	Logistic	2 categories
Cardiovascular risk stratification [‡]	1992 to 2020 annually	Logistic	3 categories with 2 dummy variables
Antihypertensive drug	1992 to 2020 annually	Logistic	2 categories
Cholesterol-lowering drugs	1992 to 2020 annually	Logistic	2 categories
Discrete follow-up period index	-	-	Cubic polynomial function

Note: [†]All time-varying covariates were collected during 1992, 2002, 2007, and 2012 visits and were imputed for 1992-2001, 2003-2006, 2008-2011, and 2013-2020 annually using the last observation carried forward.

[‡] The cardiovascular risk stratification was assessed following the ASCVD and CVD risk algorithms recommended by the Chinese guideline for the primary prevention of cardiovascular disease, in which the 10-year risk stratification of total cardiovascular risk is the same as the atherosclerotic cardiovascular disease in most cases. Beirfly, a 3-step evaluation procedure was employed: (1) participants with diabetes (aged≥40 years) or LDL-C≥4.9 mmol/L (or total cholesterol [TC]≥7.2 mmol/L) were directly classified at high risk; (2) participants who do not meet procedure (1), sex-spexific 10-year ASCVD risk assessment algorithms, including LDL-C or TC levels, hypertension, smoking status, HDL-C, and age ≥45/55 years (male/female), were used based on which 10-year ASCVD risk <5%, 5%−9%, and ≥10% were defined as low, intermediate, and high risk, respectively; (3) for those intermediate-risk participants aged <55 years, the lifetime risk of cardivascular disease were assessed, in which participants with ≥2 following risk factors are defined at high risk: a) systolic blood pressure ≥160 mmHg or diastolic blood pressure ≥100 mmHg; b) non-HDL-C ≥5.2 mmol/L (200 mg/dL); c) HDL-C <1.0 mmol/L (40 mg/dL); d) body mass index (BMI) ≥28kg/m2; and e) smoking.

Supplementary Table 2. Estimated parametric g-formula risk (%), risk difference (RD, %), and restricted mean event-free based number needed to treat (NNT) to prevent one cardiovascular disease, all-cause mortality, and atherosclerotic cardiovascular disease under natural course and treat-to-target interventions recommended by the 2020 Chinese Society of Cardiology (unless stated) after 29 years of follow-up from the Chinese Multi-provincial Cohort Study 1992-2020

**Interventions**	**Cardiovascular disease**			**All-cause mortality**			**Atherosclerotic cardiovascular disease**		
	**Risk (%, 95% CI)**	**RD (%, 95% CI)**	**NNT**	**Risk (%, 95% CI)**	**RD (%, 95% CI)**	**NNT**	**Risk (%, 95% CI)**	**RD (%, 95% CI)**	**NNT**
**Natural course**	18.6 (17.7 to 19.9)	Reference	-	25.6 (24.7 to 27.4)	Reference	-	17.7 (16.9 to 19.0)	Reference	-
**Treat-to-target interventions** †	16.3 (15.4 to 18.3)	-2.3 (-3.4 to -0.8)	115	22.7 (21.8 to 25.2)	-3.0 (-4.3 to -1.8)	95	15.1 (14.2 to 17.2)	-2.6 (-3.5 to -1.2)	104
**Subgroup analyses**									
Sex									
Women	14.4 (11.2 to 17.1)	-2.5 (-5.2 to 0.0)	112	19.5 (15.2 to 22.9)	-2.9 (-6.3 to 0.2)	102	13.2 (10.2 to 15.7)	-2.9 (-5.4 to -0.0)	99
Men	19.2 (16.6 to 22.6)	-0.8 (-3.3 to 2.3)	336	28.9 (25.5 to 32.6)	-1.6 (-4.4 to 1.2)	166	18.1 (15.5 to 21.4)	-1.0 (-3.6 to 1.8)	247
Body mass index (kg/m^2)									

<24	12.1 (10.0 to 15.2)	-2.9 (-5.2 to 0.5)	91	17.6 (14.2 to 21.2)	-4.6 (-7.8 to -1.0)	62	11.6 (9.7 to 14.8)	-2.6 (-4.9 to 1.0)	99
≥24	22.5 (19.4 to 25.0)	-1.0 (-4.4 to 1.5)	252	30.0 (25.9 to 34.1)	-0.7 (-3.5 to 3.5)	367	20.5 (17.7 to 22.9)	-2.0 (-5.1 to 0.2)	134
Smoking status									
Yes	20.8 (17.6 to 27.3)	-3.5 (-6.0 to 1.0)	75	32.5 (26.1 to 41.6)	-3.0 (-8.2 to 2.7)	88	19.9 (16.7 to 25.6)	-3.7 (-5.9 to 0.4)	73
No	16.3 (13.9 to 19.3)	-1.0 (-2.8 to 1.6)	271	21.4 (18.6 to 24.2)	-2.0 (-4.5 to 0.6)	139	15.2 (12.9 to 18.3)	-1.3 (-3.2 to 1.4)	211
Antihypertensive medication									
Yes	35.7 (26.2 to 42.0)	2.8 (-2.5 to 10.0)	-58	48.3 (36.5 to 56.8)	6.1 (1.6 to 12.8)	-31	34.1 (24.8 to 39.7)	2.0 (-3.5 to 9.0)	-76
No	16.1 (14.0 to 19.0)	-2.1 (-3.5 to 0.6)	129	21.5 (19.0 to 24.4)	-3.7 (-5.7 to -1.4)	77	14.9 (13.2 to 17.5)	-2.4 (-3.8 to 0.1)	116
**Sensitivity analyses**									
Reordering time-varying variables	16.3 (15.4 to 18.3)	-2.3 (-3.4 to -0.8)	116	22.7 (21.8 to 25.2)	-3.0 (-4.3 to -1.8)	93	15.1 (14.2 to 17.1)	-2.6 (-3.5 to -1.2)	104

Adherence rate of 70%	16.5 (15.7 to 18.6)	-2.0 (-3.0 to -0.6)	131	22.9 (22.2 to 25.4)	-2.7 (-4.0 to -1.6)	104	15.4 (14.5 to 17.4)	-2.3 (-3.1 to -1.0)	118
Adherence rate of 60%	16.7 (15.9 to 18.6)	-1.9 (-2.8 to -0.5)	142	23.0 (22.3 to 25.5)	-2.6 (-3.9 to -1.5)	110	15.6 (14.7 to 17.5)	-2.2 (-2.9 to -0.8)	127
Adherence rate of 50%	16.8 (16.0 to 18.8)	-1.7 (-2.6 to -0.4)	155	23.2 (22.5 to 25.5)	-2.4 (-3.7 to -1.4)	117	15.8 (14.8 to 17.6)	-2.0 (-2.7 to -0.7)	139
Adherence rate of 40%	17.0 (16.2 to 18.9)	-1.5 (-2.3 to -0.3)	175	23.4 (22.6 to 25.7)	-2.3 (-3.5 to -1.3)	128	16.0 (15.0 to 17.8)	-1.8 (-2.5 to -0.6)	157
Adherence rate of 30%	17.3 (16.4 to 19.1)	-1.3 (-2.0 to -0.1)	210	23.6 (22.8 to 25.8)	-2.0 (-3.1 to -1.2)	145	16.2 (15.3 to 18.0)	-1.5 (-2.2 to -0.4)	188
Adherence rate of 20%	17.6 (16.7 to 19.3)	-1.0 (-1.7 to 0.0)	279	24.0 (23.1 to 26.0)	-1.7 (-2.7 to -0.9)	179	16.6 (15.7 to 18.2)	-1.1 (-1.8 to -0.2)	250
**Positive control analyses**									
Per 1 mmol/L LDL-C reduction	15.5 (12.9 to 18.2)	-3.1 (-6.1 to -0.9)	79	21.2 (17.9 to 24.8)	-4.5 (-8.2 to -2.3)	58	13.8 (11.4 to 16.5)	-3.9 (-6.7 to -1.8)	65
Statin therapies ‡	19.0 (14.8 to 22.8)	-2.4 (-5.5 to 1.2)	112	23.7 (19.4 to 27.4)	-4.8 (-7.8 to -0.6)	57	17.6 (13.8 to 20.8)	-3.0 (-5.9 to 0.3)	92

Note: [†] Treat-to-target cholesterol-lowering intervention is based on cholesterol-lowering targets recommended by the Chinese Society of Cardiology in 2020 on LDL-C and non-HDL-C levels, i.e., for participants with diabetes at high cardiovascular risk, lower the LDL-C to <1.8 mmol/L (70 mg/dL, i.e., a fixed level drawed from a uniform distribution with a upper bound of 1.8 mmol/L) or LDL-C reduction to >50% from baseline whichever is the lowest and non-HDL-C to <2.6 mmol/L (100 mg/dL, i.e., a fixed level drawed from a uniform distribution with a upper bound of 2.6 mmol/L); for participants without diabetes who are at moderate-to-high cardiovascular risk lower the LDL-C to <2.6 mmol/L (100 mg/dL, i.e., a fixed level drawed from a uniform distribution with a upper bound of 2.6 mmol/L) and non-HDL-C to <3.4 mmol/L (130 mg/dL, i.e., a fixed level drawed from a uniform distribution with a upper bound of 3.4 mmol/L); for participants at low cardiovascular risk, lower LDL-C to <3.4 mmol/L (130 mg/dL, i.e., a fixed level drawed from a uniform distribution with a upper bound of 3.4 mmol/L) and a non-HDL-C <4.2 mmol/L (160 mg/dL, i.e., a fixed level drawed from a uniform distribution with a upper bound of 4.2 mmol/L).

[‡] The effects of statin therapies are based on the target trial emulation among participants with baseline LDL-C≥1.8 mmol/L, in which statin therapies are supposed to reduce LDL-C levels by 40% compared to the baseline LDL-C levels based on previous studies (Brandts *et al*. *The Lancet Regional Health – Europe* 2023;31:100665; Toth *et al*. *J Am Heart Assoc* 2022;11:e025551). Consequently, the observed 29-year cumulative risks of cardiovascular disease, all-cause mortality, and atherosclerotic cardiovascular risk were 21.4% (20.1% to 22.6%), 28.5% (26.8% to 29.5%), and 20.6% (19.3% to 21.6%), respectively.

Supplementary Table 3. Estimated risk ratio (RR), restricted mean event-free time (RMET, years), and cumulative percentage of intervention under treat-to-target interventions recommended by the 2020 Chinese Society of Cardiovascular compared to the natural course of no interventions after 29-year follow-up from the Chinese Multi-provincial Cohort Study 1992-2020 *via* the parametric g-formula

Interventions	Cardiovascular disease			All-cause mortality			Atherosclerotic cardiovascular disease		
	**RR (95% CI)**	**RMET (years)**	**Average % intervention**	**RR (95% CI)**	**RMET (years)**	**Average % intervention**	**RR (95% CI)**	**RMET (years)**	**Average % intervention**
**Natural course**	Reference	27.00	-	Reference	26.52	-	Reference	27.13	-
**Treat-to-target interventions** [†]	0.88 (0.82 to 0.96)	27.23	23	0.88 (0.84 to 0.93)	26.80	23	0.85 (0.81 to 0.93)	27.39	23
**Subgroup analyses**									
Sex									
Women	0.85 (0.69 to 1.00)	27.51	25	0.87 (0.71 to 1.01)	27.17	25	0.82 (0.66 to 1.00)	27.67	25
Men	0.96 (0.84 to 1.12)	26.74	20	0.95 (0.86 to 1.04)	26.08	20	0.95 (0.81 to 1.10)	26.91	20
Body mass index (kg/m^2)									
<24	0.81 (0.66 to 1.03)	27.70	23	0.79 (0.65 to 0.95)	27.36	23	0.81 (0.67 to 1.07)	27.77	23

≥24	0.96 (0.82 to 1.07)	26.57	23	0.98 (0.88 to 1.12)	26.03	23	0.91 (0.79 to 1.01)	26.85	23
Smoking status									
Yes	0.86 (0.75 to 1.04)	26.72	19	0.91 (0.76 to 1.07)	25.88	19	0.84 (0.74 to 1.02)	26.88	19
No	0.94 (0.84 to 1.09)	27.24	25	0.91 (0.81 to 1.02)	26.91	25	0.92 (0.81 to 1.08)	27.39	25
Antihypertensive medication									
Yes	1.09 (0.93 to 1.32)	24.32	16	1.14 (1.04 to 1.33)	23.26	16	1.06 (0.90 to 1.30)	24.56	16
No	0.88 (0.80 to 1.03)	27.30	21	0.85 (0.78 to 0.95)	26.97	21	0.86 (0.78 to 1.01)	27.46	21
**Sensitivity analyses**									
Reordering time-varying variables	0.88 (0.82 to 0.96)	27.23	23	0.88 (0.84 to 0.93)	26.80	23	0.85 (0.81 to 0.93)	27.39	23
Adherence rate of 70%	0.89 (0.84 to 0.97)	27.20	20	0.89 (0.85 to 0.94)	26.77	20	0.87 (0.83 to 0.95)	27.36	20

Adherence rate of 60%	0.90 (0.85 to 0.97)	27.19	19	0.90 (0.85 to 0.94)	26.76	19	0.88 (0.84 to 0.95)	27.34	19
Adherence rate of 50%	0.91 (0.86 to 0.98)	27.17	17	0.90 (0.86 to 0.95)	26.74	17	0.89 (0.85 to 0.96)	27.32	17
Adherence rate of 40%	0.92 (0.88 to 0.98)	27.15	15	0.91 (0.87 to 0.95)	26.72	15	0.90 (0.86 to 0.97)	27.30	15
Adherence rate of 30%	0.93 (0.89 to 0.99)	27.13	12	0.92 (0.88 to 0.96)	26.70	12	0.92 (0.88 to 0.98)	27.27	12
Adherence rate of 20%	0.95 (0.91 to 1.00)	27.09	9	0.93 (0.90 to 0.96)	26.67	9	0.94 (0.90 to 0.99)	27.24	9
**Positive control analyses**									
Per 1mmol/L LDL-C reduction	0.83 (0.68 to 0.95)	27.34	100	0.83 (0.69 to 0.91)	26.98	100	0.78 (0.63 to 0.90)	27.55	100
Statin therapies [‡]	0.89 (0.74 to 1.06)	27.00	100	0.83 (0.72 to 0.98)	26.80	100	0.86 (0.70 to 1.01)	27.20	100

Note: [†] Treat-to-target cholesterol-lowering intervention is based on cholesterol-lowering targets recommended by the Chinese Society of Cardiology in 2020 on LDL-C and non-HDL-C levels, i.e., for participants with diabetes at high cardiovascular risk, lower the LDL-C to <1.8 mmol/L (70 mg/dL, i.e., a fixed level drawed from a uniform distribution with a upper bound of 1.8 mmol/L) or LDL-C reduction to >50% from

baseline whichever is the lowest and non-HDL-C to <2.6 mmol/L (100 mg/dL, i.e., a fixed level drawed from a uniform distribution with a upper bound of 2.6 mmol/L); for participants without diabetes who are at moderate-to-high cardiovascular risk lower the LDL-C to <2.6 mmol/L (100 mg/dL, i.e., a fixed level drawed from a uniform distribution with a upper bound of 2.6 mmol/L) and non-HDL-C to <3.4 mmol/L (130 mg/dL, i.e., a fixed level drawed from a uniform distribution with a upper bound of 3.4 mmol/L); for participants at low cardiovascular risk, lower LDL-C to <3.4 mmol/L (130 mg/dL, i.e., a fixed level drawed from a uniform distribution with a upper bound of 3.4 mmol/L) and a non-HDL-C <4.2 mmol/L (160 mg/dL, i.e., a fixed level drawed from a uniform distribution with a upper bound of 4.2 mmol/L).

[‡] The effects of statin therapies are based on the target trial emulation among participants with baseline LDL-C≥1.8 mmol/L, in which statin therapies are supposed to reduce LDL-C levels by 40% compared to the baseline LDL-C levels based on previous studies (Brandts *et al*. *The Lancet Regional Health – Europe* 2023;31:100665; Toth *et al*. *J Am Heart Assoc* 2022;11:e025551). Consequently, the observed 29-year cumulative risks of cardiovascular disease, all-cause mortality, and atherosclerotic cardiovascular risk were 21.4% (20.1% to 22.6%), 28.5% (26.8% to 29.5%), and 20.6% (19.3% to 21.6%), respectively.

Supplementary Table 4. Estimated parametric g-formula risk (%), risk difference (RD, %), and restricted mean event-free based number needed to treat (NNT) to prevent one cardiovascular disease, all-cause mortality, and atherosclerotic cardiovascular disease under natural course and feasible interventions (unless stated) after 29 years of follow-up from the Chinese Multi-provincial Cohort Study 1992-2020

**Interventions**	**Cardiovascular disease**			**All-cause mortality**			**Atherosclerotic cardiovascular disease**		
	**Risk (%, 95% CI)**	**RD (%, 95% CI)**	**NNT**	**Risk (%, 95% CI)**	**RD (%, 95% CI)**	**NNT**	**Risk (%, 95% CI)**	**RD (%, 95% CI)**	**NNT**
**Natural course**	18.6 (17.7 to 19.9)	Reference	-	25.6 (24.7 to 27.4)	Reference	-	17.7 (16.9 to 19.0)	Reference	-
**Feasible interventions** †	16.4 (15.6 to 18.5)	-2.1 (-3.1 to -0.7)	124	22.8 (22.0 to 25.3)	-2.8 (-4.1 to -1.7)	100	15.3 (14.4 to 17.3)	-2.4 (-3.3 to -1.1)	112
**Subgroup analyses**									
Sex									
Women	14.6 (11.4 to 17.2)	-2.3 (-4.9 to 0.1)	122	19.6 (15.6 to 23.1)	-2.7 (-5.9 to 0.3)	109	13.4 (10.5 to 15.8)	-2.7 (-5.1 to 0.1)	108
Men	19.3 (16.7 to 22.5)	-0.7 (-3.2 to 2.2)	376	29.0 (25.6 to 32.6)	-1.5 (-4.2 to 1.2)	176	18.3 (15.6 to 21.3)	-0.9 (-3.5 to 1.7)	275
Body mass index (kg/m^2)									

<24	12.3 (10.2 to 15.4)	-2.7 (-5.0 to 0.6)	98	17.8 (14.4 to 21.2)	-4.4 (-7.5 to -0.9)	65	11.8 (9.9 to 14.9)	-2.5 (-4.7 to 1.1)	108
≥24	22.7 (19.7 to 25.2)	-0.9 (-4.1 to 1.5)	312	30.2 (26.0 to 34.1)	-0.6 (-3.3 to 3.5)	467	20.7 (18.0 to 23.2)	-1.7 (-4.8 to 0.3)	154
Smoking status									
Yes	21.0 (17.8 to 27.3)	-3.2 (-5.7 to 1.0)	81	32.7 (26.5 to 41.6)	-2.9 (-7.8 to 2.8)	95	20.1 (16.9 to 25.7)	-3.4 (-5.6 to 0.5)	79
No	16.4 (14.1 to 19.2)	-0.9 (-2.6 to 1.5)	306	21.5 (18.7 to 24.2)	-1.9 (-4.3 to 0.6)	148	15.3 (13.1 to 18.2)	-1.2 (-3.0 to 1.3)	235
Antihypertensive medication									
Yes	35.8 (26.3 to 41.8)	2.9 (-2.2 to 9.3)	-57	48.2 (36.3 to 56.4)	6.0 (1.5 to 12.2)	-31	34.3 (24.9 to 39.6)	2.2 (-3.1 to 8.4)	-73
No	16.3 (14.2 to 19.1)	-1.9 (-3.2 to 0.7)	142	21.7 (19.2 to 24.5)	-3.5 (-5.4 to -1.2)	82	15.1 (13.3 to 17.7)	-2.2 (-3.5 to 0.2)	127
**Sensitivity analyses**									
Reordering time-varying variables	16.5 (15.6 to 18.5)	-2.1 (-3.1 to -0.7)	127	22.8 (22.0 to 25.3)	-2.8 (-4.1 to -1.7)	100	15.3 (14.4 to 17.3)	-2.4 (-3.2 to -1.1)	114

Note: † Feasible treat-to-target cholesterol-lowering intervention, defined as 80% of eligible participants receiving the intervention at the follow-up examination over the study period, where the treat-to-target cholesterol-lowering intervention is based on cholesterol-lowering targets recommended by the Chinese Society of Cardiology in 2020 on LDL-C and non-HDL-C levels, i.e., for participants with diabetes at high cardiovascular risk, lower the LDL-C to <1.8 mmol/L (70 mg/dL, i.e., a fixed level drawed from a uniform distribution with a upper bound of 1.8 mmol/L) or LDL-C reduction to >50% from baseline whichever is the lowest and non-HDL-C to <2.6 mmol/L (100 mg/dL, i.e., a fixed level drawed from a uniform distribution with a upper bound of 2.6 mmol/L); for participants without diabetes who are at moderate-to-high cardiovascular risk lower the LDL-C to <2.6 mmol/L (100 mg/dL, , i.e., a fixed level drawed from a uniform distribution with a upper bound of 2.6 mmol/L) and non-HDL-C to <3.4 mmol/L (130 mg/dL, i.e., a fixed level drawed from a uniform distribution with a upper bound of 3.4 mmol/L); for participants at low cardiovascular risk, lower LDL-C to <3.4 mmol/L (130 mg/dL, i.e., a fixed level drawed from a uniform distribution with a upper bound of 3.4 mmol/L) and a non-HDL-C <4.2 mmol/L (160 mg/dL, i.e., a fixed level drawed from a uniform distribution with a upper bound of 4.2 mmol/L).

Supplementary Table 5. Estimated risk ratio (RR), restricted mean event-free time (RMET, years), and cumulative percentage of intervention under feasible interventions compared to the natural course of no interventions after 29-year follow-up from the Chinese Multi-provincial Cohort Study 1992-2020 *via* the parametric g-formula

Interventions	Cardiovascular disease			All-cause mortality			Atherosclerotic cardiovascular disease		
	RR (95% CI)	RMET (years)	Average % intervention	RR (95% CI)	RMET (years)	Average % intervention	RR (95% CI)	RMET (years)	Average % intervention
**Natural course**	Reference	27.00	-	Reference	26.52	-	Reference	27.13	-
**Feasible interventions** †	0.88 (0.84 to 0.96)	27.21	21	0.89 (0.84 to 0.94)	26.78	21	0.86 (0.82 to 0.94)	27.37	21
**Subgroup analyses**									
Sex									
Women	0.87 (0.70 to 1.01)	27.49	23	0.88 (0.72 to 1.01)	27.16	23	0.83 (0.68 to 1.01)	27.64	23
Men	0.97 (0.84 to 1.11)	26.73	18	0.95 (0.86 to 1.04)	26.07	18	0.95 (0.82 to 1.09)	26.90	18
Body mass index (kg/m^2)									
<24	0.82 (0.67 to 1.04)	27.68	21	0.80 (0.66 to 0.96)	27.34	21	0.83 (0.68 to 1.08)	27.74	21

≥24	0.96 (0.83 to 1.07)	26.55	21	0.98 (0.89 to 1.12)	26.02	21	0.92 (0.80 to 1.01)	26.82	21
Smoking status									
Yes	0.87 (0.76 to 1.04)	26.70	17	0.92 (0.77 to 1.07)	25.86	17	0.85 (0.75 to 1.02)	26.86	17
No	0.95 (0.85 to 1.09)	27.23	23	0.92 (0.82 to 1.02)	26.90	23	0.93 (0.82 to 1.08)	27.38	23
Antihypertensive medication									
Yes	1.09 (0.94 to 1.30)	24.32	14	1.14 (1.04 to 1.31)	23.28	14	1.07 (0.91 to 1.28)	24.54	14
No	0.89 (0.82 to 1.04)	27.28	20	0.86 (0.79 to 0.95)	26.95	20	0.87 (0.79 to 1.01)	27.44	20
**Positive control analyses**									
Reordering time-varying variables	0.89 (0.84 to 0.96)	27.21	21	0.89 (0.84 to 0.94)	26.78	21	0.87 (0.82 to 0.94)	27.37	21

Note: † Feasible treat-to-target cholesterol-lowering intervention, defined as 80% of eligible participants receiving the intervention at the follow-up examination over the study period, where treat-to-target cholesterol-lowering intervention is based on cholesterol-lowering targets recommended by the Chinese Society of Cardiology in 2020 on LDL-C and non-HDL-C levels, i.e., for participants with diabetes at high

cardiovascular risk, lower the LDL-C to <1.8 mmol/L (70 mg/dL, i.e., a fixed level drawed from a uniform distribution with a upper bound of 1.8 mmol/L) or LDL-C reduction to >50% from baseline whichever is the lowest and non-HDL-C to <2.6 mmol/L (100 mg/dL, i.e., a fixed level drawed from a uniform distribution with a upper bound of 2.6 mmol/L); for participants without diabetes who are at moderate-to-high cardiovascular risk lower the LDL-C to <2.6 mmol/L (100 mg/dL, i.e., a fixed level drawed from a uniform distribution with a upper bound of 2.6 mmol/L) and non-HDL-C to <3.4 mmol/L (130 mg/dL, i.e., a fixed level drawed from a uniform distribution with a upper bound of 3.4 mmol/L); for participants at low cardiovascular risk, lower LDL-C to <3.4 mmol/L (130 mg/dL, i.e., a fixed level drawed from a uniform distribution with a upper bound of 3.4 mmol/L) and a non-HDL-C <4.2 mmol/L (160 mg/dL, i.e., a fixed level drawed from a uniform distribution with a upper bound of 4.2 mmol/L).

Supplementary Figures

Supplementary Figure 1. The observed (red dot) and predicted (blue line) levels of low-density lipoprotein cholesterol (LDL-C) using the generalized linear mixed model after accounting for age, sex, body mass index, hypertension, diabetes, and the use of cholesterol-lowering drugs for nine randomly selected participants (IDs 661, 1842, 1895, 3723, 4857, 4893, 15730, 35986, and 35993)

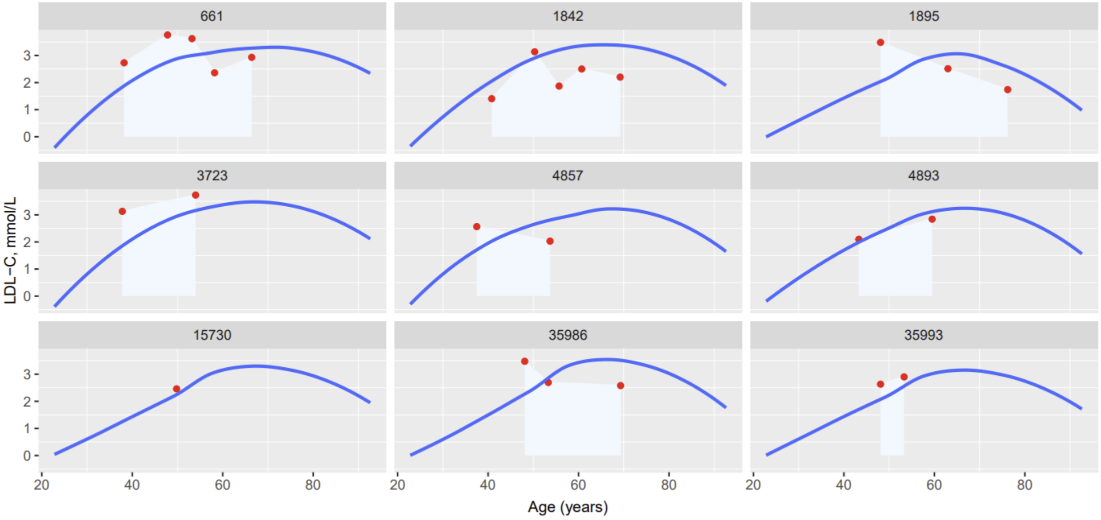

Supplement: Supplementary file 1 — Supplementary information [file 41467_2024_54078_MOESM1_ESM.pdf]
