# Supplementary material for: Effectiveness of treat-to-target cholesterol-lowering interventions on cardiovascular disease and all-cause mortality risk in the community-dwelling population: a target trial emulation
Source: Nat Commun. 2024 Nov 15;15:9922. doi: 10.1038/s41467-024-54078-4 (PMC11568141; doi:10.1038/s41467-024-54078-4)
Supplement: Supplementary file 2 — Reporting Summary [file 41467_2024_54078_MOESM2_ESM.pdf]

nature portfolio

Corresponding author(s): Jing Liu

Last updated by author(s): Sep 27, 2024

Reporting Summary

Nature Portfolio wishes to improve the reproducibility of the work that we publish. This form provides structure for consistency and transparency in reporting. For further information on Nature Portfolio policies, see our Editorial Policies and the Editorial Policy Checklist.

Statistics

For all statistical analyses, confirm that the following items are present in the figure legend, table legend, main text, or Methods section.

n/a	Confirmed	
☐	☒	The exact sample size (*n*) for each experimental group/condition, given as a discrete number and unit of measurement
☐	☒	A statement on whether measurements were taken from distinct samples or whether the same sample was measured repeatedly
☐	☒	The statistical test(s) used AND whether they are one- or two-sided<br>*Only common tests should be described solely by name; describe more complex techniques in the Methods section.*
☐	☒	A description of all covariates tested
☐	☒	A description of any assumptions or corrections, such as tests of normality and adjustment for multiple comparisons
☐	☒	A full description of the statistical parameters including central tendency (e.g. means) or other basic estimates (e.g. regression coefficient) AND variation (e.g. standard deviation) or associated estimates of uncertainty (e.g. confidence intervals)
☐	☒	For null hypothesis testing, the test statistic (e.g. *F*, *t*, *r*) with confidence intervals, effect sizes, degrees of freedom and *P* value noted<br>*Give P values as exact values whenever suitable.*
☒	☐	For Bayesian analysis, information on the choice of priors and Markov chain Monte Carlo settings
☒	☐	For hierarchical and complex designs, identification of the appropriate level for tests and full reporting of outcomes
☐	☒	Estimates of effect sizes (e.g. Cohen's *d*, Pearson's *r*), indicating how they were calculated

Our web collection on statistics for biologists contains articles on many of the points above.

Software and code

Policy information about availability of computer code

Data collection	No software was used for data collection.
Data analysis	We conducted all analyses using R 4.3.0 using gfoRmula and nnt packages. The sample code used for this study is publicly available on Github: http://github.com/yangzhao98/dynamicIntervention (DOI: https://doi.org/10.5281/zenodo.13844279).

For manuscripts utilizing custom algorithms or software that are central to the research but not yet described in published literature, software must be made available to editors and reviewers. We strongly encourage code deposition in a community repository (e.g. GitHub). See the Nature Portfolio guidelines for submitting code & software for further information.

Data

Policy information about availability of data

All manuscripts must include a data availability statement. This statement should provide the following information, where applicable:

- Accession codes, unique identifiers, or web links for publicly available datasets
- A description of any restrictions on data availability
- For clinical datasets or third party data, please ensure that the statement adheres to our policy

The data are currently private due to legal and regulatory issues. However, we welcome collaborations and research proposals. Please contact the corresponding author, Professor Jing Liu (jingliu@ccmu.edu.cn), directly.

Research involving human participants, their data, or biological material

Policy information about studies with human participants or human data. See also policy information about sex, gender (identity/presentation), and sexual orientation and race, ethnicity and racism.

Reporting on sex and gender	To identify vulnerable participants for whom the treat-to-target interventions may confer more benefit, subgroup analyses by sex (men and women). We found the protective effects of treat-to-target cholesterol-lowering interventions were more pronounced in women.
Reporting on race, ethnicity, or other socially relevant groupings	To adjust for potential confounders in Step 1, we specified a set of modified cardiovascular risk factors based on previous studies. Extended Data Figure 1 depicts the assumed causal relationships of treat-to-target cholesterol-lowering interventions with CVD, all-cause mortality, and ASCVD at each follow-up point in the presence of competing events. Specifically, we selected age at baseline and sex as time-fixed confounders, and cholesterol levels of LDL-C, non-HDL-C, cholesterol-lowering treatment, body mass index (BMI), systolic blood pressure (SBP), triglyceride (TG), smoking status, diabetes status, cardiovascular risk status, and use of antihypertensive drugs are considered time-varying confounders, of which cholesterol levels of LDL-C, non-HDL-C, cholesterol-lowering treatment were regarded as treat-to-target intervention variables.
Population characteristics	Overall, a total of 5,735 eligible CMCS participants aged ≥35 years, with no prior CVD history and no cholesterol levels missing at baseline during 1992-1993 were included. Table 2 shows the characteristics of eligible CMCS participants. The mean age at baseline was 47 years, and 61% were women. The mean LDL-C and non-HDL-C at baseline were 2.7 mmol/L and 3.3 mmol/L, respectively, with 1.4% taking cholesterol-lowering medications and 8.9% at high risk of developing ASCVD. Over the study period from 1992 to 2020, the mean LDL-C and non-HDL-C increased, along with a remarkably increased proportion of taking cholesterol-lowering medications and a higher proportion of participants at intermediate-to-high cardiovascular risk.
Recruitment	This study initially included 5,966 participants aged 35 years or older from the Chinese Multi-provincial Cohort Study recruited at the baseline during 1992-1993 (W_0). Details of the CMCS have been previously published.
Ethics oversight	This study was approved by the ethics committee of Beijing Anzhen Hospital, Capital Medical University (reference no. ks2019029). All participants provided informed consent.

Note that full information on the approval of the study protocol must also be provided in the manuscript.

Field-specific reporting

Please select the one below that is the best fit for your research. If you are not sure, read the appropriate sections before making your selection.

☒ Life sciences ☐ Behavioural & social sciences ☐ Ecological, evolutionary & environmental sciences

For a reference copy of the document with all sections, see nature.com/documents/nr-reporting-summary-flat.pdf

Life sciences study design

All studies must disclose on these points even when the disclosure is negative.

Sample size	The available information from the Chinese Multi-provincial Cohort Study determined the sample size used in the current analyses.
Data exclusions	We excluded 231 participants, including 106 with prevalent CVD, and 125 with TG ≥4.52 mmol/L (400 mg/dL) at W_0, leaving 5,735 participants available in the final analysis.
Replication	n/a
Randomization	Unlike the static intervention implemented at the baseline, treat-to-target interventions are dynamic, and participants can receive the corresponding intervention when the risk-based conditions are met during follow-up in-person examinations over the study period. Thus, we emulated the treatment strategies by transforming the follow-up time into a one-year unit and initiating the intervention when the conditions were met. We defined the start of the follow-up period (i.e., time zero) as the initiation time of the intervention when risk-based conditions were met. We stopped the cholesterol-lowering interventions immediately after the cholesterol-lowering targets were met.
Blinding	n/a

Reporting for specific materials, systems and methods

We require information from authors about some types of materials, experimental systems and methods used in many studies. Here, indicate whether each material, system or method listed is relevant to your study. If you are not sure if a list item applies to your research, read the appropriate section before selecting a response.

Materials & experimental systems

n/a	Involved in the study
☒	☐ Antibodies
☒	☐ Eukaryotic cell lines
☒	☐ Palaeontology and archaeology
☒	☐ Animals and other organisms
☒	☐ Clinical data
☒	☐ Dual use research of concern
☒	☐ Plants

Methods

n/a	Involved in the study
☒	☐ ChIP-seq
☒	☐ Flow cytometry
☒	☐ MRI-based neuroimaging

Plants

Seed stocks

Report on the source of all seed stocks or other plant material used. If applicable, state the seed stock centre and catalogue number. If plant specimens were collected from the field, describe the collection location, date and sampling procedures.

Novel plant genotypes

Describe the methods by which all novel plant genotypes were produced. This includes those generated by transgenic approaches, gene editing, chemical/radiation-based mutagenesis and hybridization. For transgenic lines, describe the transformation method, the number of independent lines analyzed and the generation upon which experiments were performed. For gene-edited lines, describe the editor used, the endogenous sequence targeted for editing, the targeting guide RNA sequence (if applicable) and how the editor was applied.

Authentication

Describe any authentication procedures for each seed stock used or novel genotype generated. Describe any experiments used to assess the effect of a mutation and, where applicable, how potential secondary effects (e.g. second site T-DNA insertions, mosiacism, off-target gene editing) were examined.
